# Mixed lineage leukemia-septin 5 fusion transcript in *de novo* adult acute myeloid leukemia with t(11;22)(q23;q11.2): A case report

**DOI:** 10.3892/ol.2014.1971

**Published:** 2014-03-14

**Authors:** WEN GAO, TONG WANG, YIN WU, HONG XING LIU, YAN CHEN LI, WEN MING CHEN

**Affiliations:** 1 Department of Hematology, Myeloma Research Center of Beijing, Beijing Chaoyang Hospital, Capital Medical University, Beijing 100020, P.R. China; 2 Center of Lu Dao-Pei Hematology Neoplasm, He-Bei Yan Da Hospital, Langfang, Hebei 065201, P.R. China

**Keywords:** mixed lineage leukemia, 11q23, septin 5, septin, acute myeloid leukemia

## Abstract

The current report presents a case of *de novo* acute myeloid leukemia (AML) in a 32-year-old male. Cytogenetic analysis showed that the karyotype of the bone marrow cells was as follows: 46,XY,t(11;22)(q23;q11.2)[13]/46,X,−Y,+10,t(11;22)(q23;q11.2)[7]/47,XY,+10,t(11;22)(q23;q11.2)[1]/46,XY[1]. Fluorescence in situ hybridization analysis using a mixed lineage leukemia (MLL)-specific probe showed a split in the MLL gene. Reverse transcription polymerase chain reaction (PCR) analysis demonstrated an MLL-septin 5 (SEPT5) fusion transcript in the patient. Nucleotide sequencing analysis of the PCR product confirmed the fusion between the MLL exon 9 and SEPT5 exon 3, and the product was 521 bp in length. The present study reviewed the clinical and molecular features of the AML with an MLL-SEPT5 fusion gene.

## Introduction

Chromosomal rearrangements involving the mixed lineage leukemia (MLL) gene are the most common genetic alterations in acute leukemia, particularly in infants (1), and the majority of the rearrangements indicate a poor prognosis (2).

Reciprocal translocation represents the most frequent form of MLL rearrangement (3,4). All the chromosomal breaks occur in the 8.3-kb breakpoint cluster region within the MLL gene between introns 8 and 13 (5). To date, >60 translocation gene partners have been identified (3,4). In pediatric and adult acute myeloid leukemia (AML), the most frequent fusion partners are represented by MLLT3/AF9 (9p22), MLLT10/AF10 (10p12), ELL (19p13.1), MLLT4/AF6 (6q27) and MLLT1/ENL (19p13.3) (4), however, t(11;22)(q23;q11) is rare.

In total, eight cases of AML with 11q23 rearrangement involving the 22q11 region as the partner have previously been reported (6–12); among these cases, only one was adult AML-M2. The current report presents a new case of AML-M2 in a 32-year-old male patient and reviews previous cases in the literature. Patient provided written informed consent.

## Case report

In August 2012, a 32-year-old male was admitted to the Department of Hematology (Beijing Chaoyang Hospital, Beijing, China) with asthenia, nausea, vomiting, spontaneous ecchymosis, a cough and a fever. The patient’s hemoglobin level was 7.2 g/dl, platelet count was 13×10^9^/liter and white blood cell count was 2.44×10^9^/liter. Bone marrow smears were hypercellular containing 61.5% myeloblasts, 7% promonocytes and 2.5% monoblasts. In addition, the blasts in the bone marrow were positive for peroxidase staining. Immunophenotypic analysis revealed that the blasts were positive for cluster of differentiation (CD)117, CD33 and CD64, weakly positive for CD15, human leukocyte antigen-DR, myeloperoxidase and CD13, and negative for CD22, CD56, cCD3, CD11b, CD14, CD19, CD34 and CD7. The patient had been well prior to the development of these symptoms, had no history of exposure to organic solvents or dye, and had never received irradiation or anticancer agents.

The diagnosis was determined as AML with maturation (French-American-British classification of M2). The patient commenced a standard 7+3 schedule with cytarabine and daunorubicin as an induction therapy. Following the achievement of complete remission (CR), the patient underwent consolidation chemotherapy followed by a medium dose of cytarabine. The patient relapsed following three cycles of medium-dose cytarabine and subsequently received salvage therapy, however, the patient succumbed to a cerebral hemorrhage in March 2013.

A bone marrow sample was processed following short-term culture (24 h) according to the standard procedures. The chromosomes were stained by G-banding and the karyotype was determined according to recommendations from the International System for Human Cytogenetic Nomenclature (2009) (13).

Fluorescent *in situ* hybridization (FISH) was performed on 200 interphase cells using the Vysis LSI MLL dual color break apart translocation probes (Abbott Molecular, Inc., Des Plaines, IL, USA).

RNA was extracted from the patient’s peripheral blood cells using the Whole Blood RNA isolation kit (BioChain Institute, Inc., Newark, CA, USA). In total, 1–2 μg of RNA was reverse transcribed to cDNA using the Thermo Scientific Maxima First-Strand cDNA synthesis kit (Thermo Fisher Scientific, Waltham, MA, USA). cDNA solution (2 μl) was amplified by polymerase chain reaction (PCR) to a total volume of 20 μl with 0.5 μM of each primer and 10 μl master mix. The primers used for the first reverse transcription (RT)-PCR were as follows: Sense, TACAGGACCGCCAAGAA for MLL-5S; and antisense, TTGGGCAGCTTCACGAAGTC for SEPT-5A. The nucleotide sequences of the PCR products were determined by a BigDye Terminator v3.1 cycle sequencing kit (Invitrogen Life Technologies, Paisley, UK).

**Figure 1:**
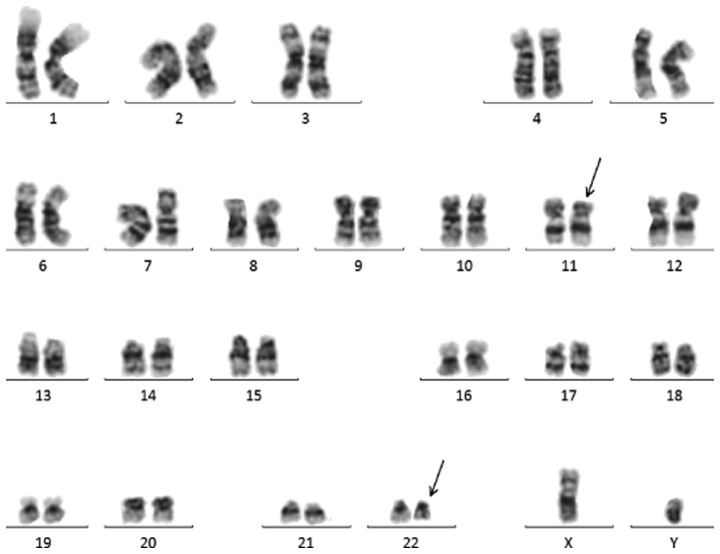
Representative karyotype of the bone marrow cells was 46,XY,t(11;22)(q23;q11.2)[13]/46,X,-Y,+10, t(11;22)(q23;q11.2)[7]/47,XY,+10,t(11;22)(q23;q11.2)[1]/46,XY[1]. Arrows indicate the derivative chromosomes 11 and 22.

**Figure 2:**
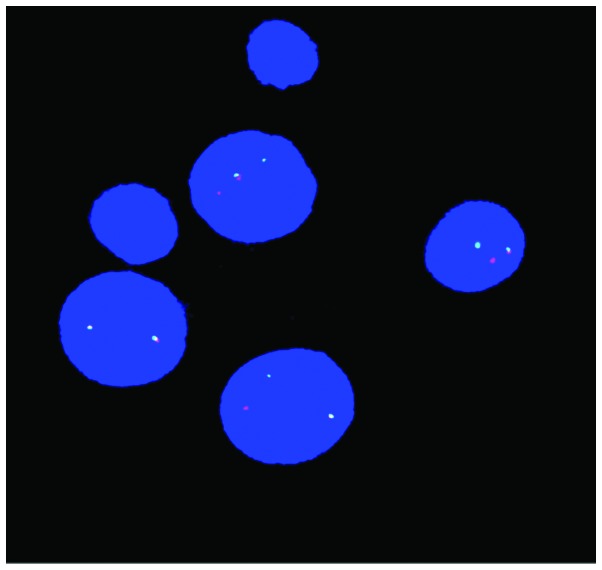
Fluorescent *in situ* hybridization analysis of the leukemic metaphase. A fusion signal between 5′ mixed lineage leukemia (MLL; red) and 3′ MLL (green) was observed on the normal chromosome 11. A split signal indicated the abnormality of MLL.

The karyotype of the bone marrow cells from the patient was identified as 46,XY,t(11;22)(q23;q11.2)[13]/46,X, −Y,+10,t(11;22)(q23;q11.2)[7]/47,XY,+10,t(11;22)(q23;q11.2)[1]/46,XY[1] in the 22 cells that were examined (Fig. 1). FISH analysis using an MLL-specific probe showed a split in the MLL gene (Fig. 2). This result indicated that the gene was involved in this translocation.

**Figure 3:**
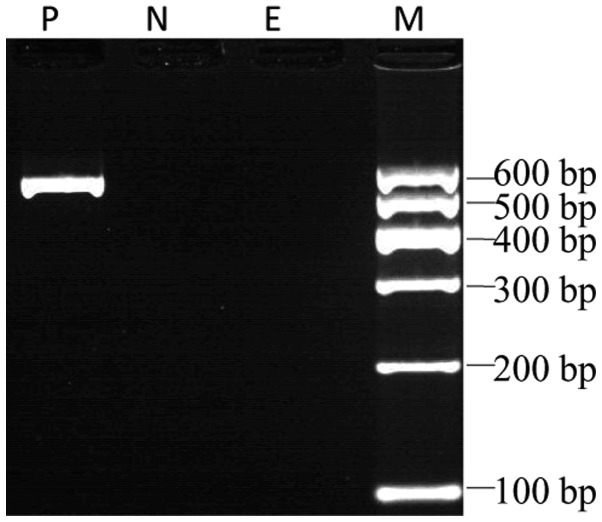
Identification of mixed lineage leukemia-septin 5 fusion transcript by reverse transcription polymerase chain reaction (PCR). Lane M, 1-kb DNA ladder; Lane P, patient sample; Lane N, PCR control; Lane E, empty control.

**Figure 4:**
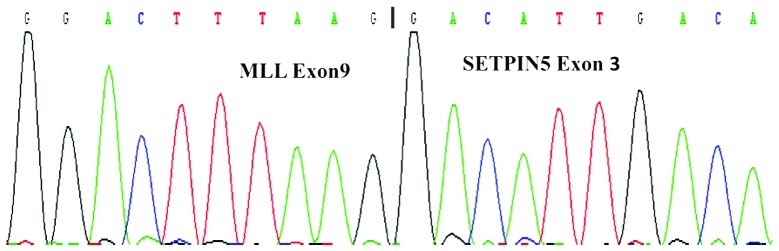
Sequence of the junction of the MLL-SEPT5 chimeric transcript showing the juxtaposition of MLL exon 9 and SEPT5 exon 3. MLL, mixed lineage leukemia; SEPT5, septin 5.

To demonstrate an MLL-septin 5 (SEPT5) fusion transcript in the current t(11;22)-AML patient, RT-PCR analysis was performed using the MLL-5S primer and an antisense primer, SEPT-5A (Fig. 3). Nucleotide sequencing analysis of the PCR product demonstrated the fusion between MLL exon 9 and SEPT5 exon 3, and the product was 521 bp in length (Fig. 4).

## Discussion

The present study describes an adult patient who presented with *de novo* AML-M2 with t(11;22)(q23;q11.2), which resulted in fusion of the MLL gene to the SEPT5 gene. The first case of adult AML-M2 with this translocation was reported in 2001 (8), and to the best of our knowledge, the current study presents the second case of reported adult AML-M2 with t(11;22). Although the patient exhibited a positive response to the induction therapy of cytarabine plus daunorubicin and achieved CR, the patient relapsed following three cycles of consolidation chemotherapy. The duration of CR was extremely short.

Several cases of AML with t(11;22)(q23;q11) have previously been reported. A 66-year-old female was diagnosed with AML-M5 with t(11;22)(q23;q11.2) and received two cycles of combination chemotherapy, including etoposide, cytosine arabinoside, vinca alkaloids and mitoxantrone (12). Remission was achieved at two months following chemotherapy, however, the CR lasted only six months. In an additional report (7), a 39-year-old male was diagnosed with AML-M2 and the karyotype of the bone marrow cells was 46,XY,t(11;22)(q23;q11) in all 20 cells that were examined. The patient was treated with idarubicin and cytarabine and achieved CR. However, the patient relapsed two months later and eventually succumbed to the disease 12 months following the first diagnosis without responding to chemotherapy; the duration of CR was only two months. In addition, a 36-year-old male was previously diagnosed with AML-M4 with t(11;22)(q23;q11) (9). The patient was treated with standard-dose cytarabine and daunorubicin and achieved CR. Although the patient accepted high-dose cytarabine as a consolidation therapy, CR was only 8.9 months and overall survival was 20.8 months. More recently, a 23-month-old female was diagnosed with AML-M5 with t(11;22)(q23;q11). The patient was enrolled in the ELAM 02 protocol (aracytine, mitoxantrone and methotrexate) and achieved CR following induction chemotherapy with mitoxantrone plus cytarabine. According to the protocol, the patient received an additional transplant of bone marrow from a sibling and the duration of CR was more than two years (6). It appeared that the allogenic bone marrow transplantation (BMT) overcame the impact of t(11;22) q23;q11).

To date, only 32 cases of AML with MLL-SEPT fusions have been reported in the literature (5,6), including four cases of MLL-SEPT5 fusion (6,8,9). The current study describes a novel case of AML with MLL-SEPT5 fusion. Molecular studies demonstrated a fusion of the MLL exon 9 and SEPT5 exon 3. The four previously reported cases of the MLL-SEPT5 rearrangement presented the following fusions: MLL exon 7 and SEPT5 exon 3 for two patients (9); MLL exon 10 and SEPT5 exon 3 for one patient (6); and MLL exon 6 and SEPT5 exon 4 for one patient (8). Although the MLL-SEPT5 fusion transcripts identified in the current study are different to those previously reported, it appeared that the SEPT5 exon 3 had a greater frequency of involvement. Further studies are required to understand the impact of such a fusion on prognosis.

In conclusion, although the AML patients with t(11;22)(q23;q11) have exhibited positive responses to the induction therapy, the duration of CR is relatively short under conventional chemotherapy. An allogenic BMT appeared to overcome the impact of t(11;22)(q23;q11), however, future studies are required to confirm this.
